# Anti-Inflammatory *Lactobacillus rhamnosus* CNCM I-3690 Strain Protects against Oxidative Stress and Increases Lifespan in *Caenorhabditis elegans*

**DOI:** 10.1371/journal.pone.0052493

**Published:** 2012-12-26

**Authors:** Gianfranco Grompone, Patricia Martorell, Silvia Llopis, Núria González, Salvador Genovés, Ana Paula Mulet, Tamara Fernández-Calero, Inés Tiscornia, Mariela Bollati-Fogolín, Isabelle Chambaud, Benoit Foligné, Agustín Montserrat, Daniel Ramón

**Affiliations:** 1 Danone Research, Gut Microbiology & Probiotics Platform, Palaiseau, France; 2 Institut Pasteur de Montevideo, Montevideo, Uruguay; 3 Department of Food Biotechnology, Biopolis S.L., Parc Científic Universitat de València, Edificio 2; C/Catedrático Agustín Escardino Benlloch, n° 9; Valencia, Spain; 4 Bioinformatics Unit, Institut Pasteur de Montevideo, Montevideo, Uruguay; 5 Cell Biology Unit, Institut Pasteur de Montevideo, Montevideo, Uruguay; 6 Bactéries Lactiques & Immunité des Muqueuses, Institut Pasteur de Lille (IPL), Center for Infection and Immunity of Lille, INSERM U 1019, Université Lille Nord de France, CNRS UMR 8204, Lille, France; 7 Danone Research Spain c/Corcega 590, Barcelona, Spain; Teagasc Food Research Centre, Ireland

## Abstract

Numerous studies have shown that resistance to oxidative stress is crucial to stay healthy and to reduce the adverse effects of aging. Accordingly, nutritional interventions using antioxidant food-grade compounds or food products are currently an interesting option to help improve health and quality of life in the elderly. Live lactic acid bacteria (LAB) administered in food, such as probiotics, may be good antioxidant candidates. Nevertheless, information about LAB-induced oxidative stress protection is scarce. To identify and characterize new potential antioxidant probiotic strains, we have developed a new functional screening method using the nematode *Caenorhabditis elegans* as host. *C. elegans* were fed on different LAB strains (78 in total) and nematode viability was assessed after oxidative stress (3 mM and 5 mM H_2_O_2_). One strain, identified as *Lactobacillus rhamnosus* CNCM I-3690, protected worms by increasing their viability by 30% and, also, increased average worm lifespan by 20%. Moreover, transcriptomic analysis of *C. elegans* fed with this strain showed that increased lifespan is correlated with differential expression of the DAF-16/insulin-like pathway, which is highly conserved in humans. This strain also had a clear anti-inflammatory profile when co-cultured with HT-29 cells, stimulated by pro-inflammatory cytokines, and co-culture systems with HT-29 cells and DC in the presence of LPS. Finally, this *Lactobacillus* strain reduced inflammation in a murine model of colitis. This work suggests that *C. elegans* is a fast, predictive and convenient screening tool to identify new potential antioxidant probiotic strains for subsequent use in humans.

## Introduction

Aerobic metabolism leads to the production of harmful byproducts. Organisms can only stay healthy by reducing natural by-products of oxygen metabolism, such as reactive oxygen species (ROS), which are mainly produced by mitochondria [1] and damage proteins, lipids and DNA on accumulating in cells [2], [3], [4], [5]. Oxidative stress plays an important role in the rate of aging processes, often referred to as the Mitochondrial Free Radical Theory of Aging [6], [7], [1]. This process is also a key factor in aging-associated degenerative diseases such as certain types of cancer, diabetes and heart failure, among others [4], [7]. Oxidative stress also plays an obvious role in the pathogenesis of a number of gastrointestinal diseases, including: gastric and duodenal ulcer disease, pancreatitis, IBD; gastric, esophageal, and colon cancers [8], [9]. Although the causal role of ROS in aging remains controversial, recent reports suggest that ROS mediate a stress response to age-related damage, rather than being the underlying cause of aging [10]. Moreover, ROS are shown to have protective effects in model organisms such as *Saccharcomyces cerevisae*, *Caenorhabditis elegans* and *Drosophila melanogaster* [11].

The search for new nutritional antioxidant compounds able to counterbalance the effects of oxidative stress poses an exciting research challenge. There is increasing pre-clinical and clinical evidence that nutritional interventions, using food products or food-grade compounds, can protect against oxidative stress [12], [13], [14], [15]. Probiotics, defined by FAO/WHO in 2001 as live micro-organisms which, when administered in adequate amounts, confer a health benefit to the host [16], can be good candidates for providing such antioxidant effects. While various methodologies have been developed to identify natural LAB strains with intrinsic antioxidant properties [17], [18], there are insufficient methods available for the fast and reliable massive screening of bacterial culture collections *in vivo* using complex multicellular organisms. Indeed, only a few natural antioxidant lactic acid bacteria (LAB) have been characterized in animals so far [19]. In systems mimicking colon fermentation, *Lactobacillus paracasei* Fn032, *Lactobacillus rhamnosus* GG and *Lactobacillus* spp *Fn 001* have been shown to prevent hydroxyl radical production [20]. Moreover, it has been shown that orally-administered live recombinant LAB (*Lactococcus lactis* or *Lactobacillus plantarum* strains) producing bacterial SOD can improve TNBS-induced colitis in rats [21], [22] and mice (Foligné *et al.*, unpublished data).

However, laboratory animals cannot be used in the first steps of screening large sets of strains and, thus, there is the need for more convenient and predictable screening tools for natural antioxidant LAB. In this respect, the use of less evolved animals may provide an attractive alternative [23], [24].

To identify new natural LAB strains with antioxidant effect, we have developed a completely new and highly predictive method that uses *Caenorhabditis elegans* as an *in vivo* screening model. *Caenorhabditis elegans* is an extremely powerful and well-studied biological system, which has been used as a model to study aging and related diseases [25], [10], [26]. This nematode is a good biological model for genetic studies [27] since many of its pathways are conserved in humans. Oxidative damage and its effects on aging have been studied in a *C. elegans* model system using a nematode mutant strain exhibiting hyper-resistance to oxidative stress, as compared to its parental strain [28]. Moreover, *C. elegans* proved an appropriate model to screen potential anti-*Salmonella* infection bacteria [29] and antibacterial compounds [30]. Furthermore, *C. elegans* longevity is known to be related to the insulin pathway and to orthologous genes in humans, involved in the insulin-like growth factor and diabetes [31], [32]. So far, little is known about the potential use of *C. elegans* as a screening tool for probiotic bacteria inducing resistance against oxidative stress or improving longevity.

Here, we have used the nematode *Caenorhabditis elegans* as a new preclinical model to carry out preliminary antioxidant screenings to identify potential probiotic strains and to provide insights into the mechanisms by which these strains lower oxidative stress. This animal model has enabled us to identify a new strain of *Lactobacillus rhamnosus*, designated CNCM I-3690, which exerted a strong antioxidant effect and extended nematode lifespan through the insulin-like pathway DAF-2/DAF-16. Furthermore, since inflammation can be associated with generation of ROS leading to oxidative stress [33], we observed that CNCM I-3690 strain might have the ability to protect against oxidative stress through an anti-inflammatory effect. These findings suggest that *C. elegans* can be a good predictive screening tool for new potential probiotic strains.

## Materials and Methods

### Cultures of Lactic Acid Bacteria (LAB) and Bifidobacteria

We included 78 bacterial strains from a Danone Research collection in the *in vivo* antioxidant screening methodology, using the model organism *C. elegans*, adapted from a previously reported protocol performed to measure the antioxidant activity of plant extracts [12]. This collection is composed by 62 *Lactobacillus* strains belonging to *acidophilus*, *bulgaricus*, *casei*, *paracasei*, *plantarum* and *rhamnosus* species, 9 *Streptococcus thermophilus* isolates and 6 *Bifidobacterium* strains belonging to the *animalis*, *breve* and *longum* species. See Table S1 for genera and species specifications of each strain used in this study. The strains belonging to *Bifidobacterium*, *Lactobacillus* and *Streptococcus* genera were grown in MRS with cysteine, MRS and Elliker media, respectively. As the bioassay of the *in vivo* antioxidant activity is to be carried out with samples of live cells of different LAB, cells must be recovered in the logarithmic phase growth. After growth curve analysis, the optimal time for cell recovery was established to be after 15 h of incubation at OD600 = 1, 1.5 and 1.7 for *Streptococcus*, *Lactobacillus* and *Bifidobacterium,* respectively. The different LAB cultures were added to the mixture at a final concentration of 4×10^6^ cells/mL. See Supplementary Material S1 for growth curves of representative strains for each genus and detailed protocol.

In addition, we analyzed the sensitivity of bacterial strains to kanamycin, an antibiotic used to inhibit *Escherichia coli* growth. A concentration of 30 µg/mL was sufficient to inhibit *E. coli* OP50.

### Cultures of *Caenorhabditis Elegans*

Experiments were carried out with a *C. elegans* mutant strain BA17 *fem-1(hc17)* (*Caenorhabditis* Genetics Center at the University of Minnesota, USA) which is infertile at 25°C. BA17 worms were synchronized by isolating eggs from gravid adults at 20°C, hatching the eggs overnight in M9 buffer and isolating L1-stage worms in the wells of a microtiter plate. The worms were grown without shaking for three days at 25°C and 80–85% relative humidity. After this incubation period, adult worms were subjected to oxidative stress with H_2_O_2_ (3 mM and 5 mM) or without H_2_O_2_ (no stress control). Two controls were used during this experiment: wells with *Escherichia coli* instead of LAB as the control of bacterial feeding and wells with *E. coli* and 3 mM or 5 mM H2O2 as the control for oxidative stress. Worms were incubated in these conditions for 5 h. In terms of scoring for antioxidant capacity, we considered paralyzed worms to be dead (stressed).

### Oxidative Stress Assays on Agar Plates

In these experiments, we used the *C. elegans* wild type strain N2 (*Caenorhabditis* Genetics Center at the University of Minnesota, USA) to validate the potential antioxidant activity of a group of selected bacterial strains. For this purpose, worms were grown in NG medium (Nematode Growth medium: Agar 17.5 g/l, Sodium Chloride 3.0 g/l, Peptone 2.5 g/l, Cholesterol 0.005 g/l) on agar plates with a lawn of *E.coli* OP50 (control media) and NG with a lawn of each LAB. Worms were incubated at 20°C for 5 days, and then transferred to a medium with 3 mM of H_2_O_2_. After 5 h of incubation, worm viability was scored.

### Longevity Assays in *C. elegans*

To measure the lifespan of *C. elegans*, synchronized worms of the wild-type strain (N2) and the mutant strains LG333 (*skn-1*), GR1307 (*daf-16*) and CB1370 (*daf-2*) were grown at 20°C until they reached the young adult stage. All mutant strains of *C. elegans* were obtained from the *Caenorhabditis* Genetics Center at the University of Minnesota (USA). Worms were then transferred to NGM agar plates covered with lawns of *E. coli* OP50 or the corresponding LAB (CNCM I-3690 or CNCM I-4317). The plates were incubated at 20°C and the numbers of live and dead worms were scored every two days. Parents were moved periodically to new plates to separate them from their progeny. A worm was considered as dead if it failed to respond to a platinum wire. Three independent assays were carried out with each strain.

### Transcriptomic Analysis in *C. elegans*

Gene expression in *C. elegans* wild-type strain (N2) was analyzed in worm populations fed with *E. coli* OP50 (control condition) or the corresponding LAB (*Lactobacillus rhamnosus* CNCM I-3690 or *Lactobacillus rhamnosus* CNCM I-4317). Three days feeding period was analyzed (young-adult worms). Synchronized populations were obtained from embryos isolated from gravid adults in the different feeding conditions. After feeding period (3 days), samples of worms were collected with M9 buffer, washed three times and collected in eppendorf tubes for worm disruption by sonication (3 pulses at 10 W, 20 s/pulse). Total RNA isolation was performed with RNAasy Kit (Qiagen, Barcelona, Spain). RNA samples were processed for hybridization using the GeneChip® *C. elegans* Genome Array of Affymetrix (UCIM, University of Valencia). These chips contain oligonucleotide probesets designed to asses over 22500 transcripts from the *C. elegans* genome. Four biological replicates were examined per condition using Bioinformatics (CIPF, Valencia, Spain). Raw data obtained from Affymetrix arrays were background corrected using RMA methodology [34]. Signal intensity was standardized across arrays via quantile normalizaton algorithm. Differential gene expression assessment between control and treated conditions was carried out using limma moderated t-statistics. The p-values obtained for each gene were adjusted with multiple testing p-value correction procedures [35]. Finally, gene set analysis was carried out for each comparison using logistic regression models ner [36].

### Microarray Data Analysis

Data analysis was accomplished in “R” (R Foundation for Statistical Computing, Vienna, Austria; http://www.R-project.org) mainly through packages in the “Bioconductor” suite [37]. Normalization was performed using rma (robust multi-array average expression measure) in “affy” [38] and differential expression was assayed via “limma” [39]. Genes were considered differentially expressed when the multiple testing adjusted *P*-value <0,01. Ontology analysis was performed with “GeneAnswers” (R package version 1.8.0).

The microarray data produced and discussed in this work have been deposited in NCBI's Gene Expression Omnibus and are accessible through GEO Series accession number GSE42192 (http://www.ncbi.nlm.nih.gov/geo/query/acc.cgi?acc=GSE42192).

### RT-qPCR Experiments and Analysis

Total RNA was extracted using RNeasy (Qiagen), and reverse transcribed using RevertAid H Minus Reverse Transcriptase (Fermentas). The resulting cDNA was subjected to RT-qPCR analysis using SYBR Green detection (LightCycler® FastStart DNA MasterPLUS SYBR Green, Roche) on LightCycler (Roche). Oligonucleotides for RT-qPCR were designed using Primer3Plus. All CT values were normalized against the control gene Y45F10D.4, which did not vary under the conditions being tested. All experiments were repeated at least three times (biological replicates) and were internally controlled (technical replicate). Expression changes were obtained by calculating the relative expression levels using the 2^−ΔΔCT^ method. For expression ratio and comparison between microarray data and RT-qPCR data see Supplementary Material S8.

### Nile Red Staining

The effect of the selected lactic bacteria strains on *C. elegans* body fat reduction was studied via fluorescence measurement in Red Nile (0.05 µg/mL, Sigma, St. Louis, USA) stained nematodes. Worms of the wild type strain N2 and the strain BX153, mutant in the 9 stearoyl-CoA fatty acid desaturase FAT-7, were cultured in NGM plates in the presence of *E. coli* OP50, *Lactobacillus rhamnosus* CNCM I-3690 or *Lactobacillus rhamnosus* CNCM I-4317. Fluorescence of age-synchronized young-adult worms (3-days-old) was quantified in a VersaFluorTM Fluorometer System (Bio-Rad, Hercules, USA). A total of 180 worms per condition were analyzed. Experiments were carried out in triplicate.

### Epithelial Cell Co-cultures

HT-29 (HTB-38, ATCC) cells were cultured in DMEM supplemented with 10% fetal bovine serum. Cells were routinely propagated in 25 or 75 cm^2^ tissue culture flasks at 37°C, 5% CO2 in a humidified incubator until reaching 80–90% confluence. Subsequently, cells were trypsinized, adjusted the cellular concentration and used for different experiments. The initial passage number of the HT-29 wild-type cells was 5. Cells used for each assay were cultured for less than 15–20 passages.

HT-29 cells were seeded in a 12 well plate at a cell density of 2×10^5^/well using a final volume of 2 mL and incubated at 37°C, 5% CO2 for 24 h. Cells were transfected with the pIgK-luciferase plasmid using a DNA: Lipofectamine 2000 ratio of 1∶2.5 (µg:µL). The NF-κB reporter system by using the pIgK-luciferase plasmid was kindly provided by Philippe Sansonetti’s group at Institut Pasteur (Paris, France), and is referred in [40].

Bacteria were grown overnight at 37°C in MRS broth (Oxoid, Cambridge, UK), then subcultured, and harvested by centrifugation (5 min at 3000 g). On co-culture day, bacteria were washed twice with PBS buffer and re-suspended in DMEM. A correlation curve between A_570 nm_ and colony forming units was performed for each strain and A_570 nm_ values were used for calculating the bacterial number used in each experiments.

Twenty four hours post-transfection, media was renewed and cells were pre-incubated for 2 hours with each bacterial strain using a MOI of 100 bacteria per cell. The MOI was determined taking into account the initial cell density of 2×10^5^ cells/well. After 2 hours of pre-incubation with bacteria, an inflammatory stimulus was added to the cell without washing bacteria. The pro-inflammatory cytokine cocktail consisted of: TNF-α, IL-1β and IFN-γ; 50 ng, 2.5 ng and 7.5 ng/well, respectively and was applied for 6 hours. Viability of epithelial cells was controlled by trypan blue exclusion method. After this incubation time, supernatants were collected, cleared by centrifugation at 3000 g for 5 minutes, transferred to a new tube and finally frozen at −80°C for further cytokine quantification by Flow Cytometry and/or Luminex. Cells were rinsed with PBS and immediately lysated with Luciferase Cell Culture Lysis Reagent 1X (Promega E1500) according to the manufacturer instructions. Cell lysates were frozen at −80°C for further determination of I-κB.

### Heterotypic Co-culture Systems

Non-polarized HT-29-NF-κB-luciferase cells (passage 25–35) were grown in the upper chamber of a transwell filter (3 µm diameter of pores; Costar, USA) and incubated for 2 days in RPMI supplemented with 10% heat-inactivated FCS. Monocyte-derived DCs were generated according to a modified protocol described by Dauer *et al.* [41]. Human PBMC were obtained after Ficoll-Hypaque density-gradient centrifugation of leukoreduction system chambers of plateletpheresis from healthy donors. Briefly, monocytes from PBMC were purified by plastic adherence. For DC differentiation, monocytes were incubated for 2 days in RPMI supplemented with 10% (v/v) heat-inactivated FBS, 800 U/mL GM-CSF (Clausen, Uruguay) and IL-4 (1% of conditioned supernatant from IL-4 transfected J588L cell line). Immature DC were harvested and cultured in twelve-well tissue culture plates (1.25×10^5^ cells/well) for experiments.

Bacteria were grown overnight at 37°C in MRS broth (Oxoid, Cambridge, UK), then subcultured, and harvested by centrifugation (5 min at 3000×g). On co-culture day, bacteria were washed twice with PBS buffer and re-suspended in RPMI. A correlation curve between A_570 nm_ and colony forming units was performed for each strain and A_570 nm_ values were used for calculating the bacterial number used in each experiments. Bacteria were added apically in a 25∶1 relation (bacteria: DC). In addition to the correlation curve, the same day of each experiment, bacterial cells were inoculated in MRS plate, cultured ON and the colony number was determined. We considered valid only those experiments where a ratio of 25∶1 was achieved. Viability of epithelial cells and DC was controlled by trypan blue exclusion method.

LPS from *E. coli* O26:B6 (Sigma, Saint Louis, US) was added apically to a final concentration of 0.5 µg/mL per well. The co-culture was incubated for 18 h at 37°C in a 5% CO_2_ humidified atmosphere. HT-29-NF-κB-luciferase cells were harvested and lysed for protein and luminiscence determination and DCs surface markers were stained and analysed by flow cytometry. Co-culture supernatant was collected, cleared by centrifugation at 3000 g for 5 minutes, transferred to a new tube and finally frozen at −80°C for further cytokine quantification.

### Protein and Luminescence Determination

Protein content of the cell lysate was determined by BCA method (SIGMA, Saint Louis, US) according to the manufacturer’s instructions. For the luminescence assay, luciferase kit was used according to the manufacturer’s instructions (Promega, Madison, USA). Luciferase was quantified in a luminometer with 0.5 second of measurement per well and 0.5 second delay, at maximum gain (LUMIstar Optima, BMG).

### Cytokine Measurements

IL-8, IL-10, IL-6, TNF-α, and IL-12 concentrations were determined by FlowCytomix™ technology (Bender MedSystems, Austria) and analyzed by flow cytometry. For results calculation BMS FlowCytomix Software version 2.2.1 was used.

### Flow Cytometry and Antibodies

The following antibodies (all purchased from BD PharMingen) were used for flow cytometry stainings: B-ly6 (anti-human CD11c, allophycocyanin-conjugated), 2331 (anti-human CD86, phycoerythrin (PE)-conjugated), TU36 (anti-human HLA-DR fluorescein isothiocyanate *(*FITC)-conjugated), HIB19 (anti-human CD19 PE-conjugated), HIT3a (anti-human CD3 FITC-conjugated), M5E2 (anti-human CD14 FITC-conjugated), all with matched isotype controls. The level of expression was analyzed as the median of the fluorescence intensity (MFI). DCs were stained and then analyzed using a CyAn™ ADP (DAKO) flow cytometer and Summit 4.3 software. For each analysis 10,000 counts were recorded, gated on FSC vs SSC dot plot. For results comparison the following equation was used:
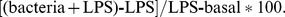



### *In vivo* Colitis Murine Mice Model

#### Ethics statement

Animal experiments were performed in an accredited establishment (number A59107; animal facility of the Institut Pasteur de Lille, France) and carried out in accordance with the guidelines of laboratory animal care published by the regional ethical committee (CREEA Nord-Pas de Calais) and subject to the rules of the European Union Normatives (number 86/609/EEC).

The name of the Institutional Animal Care and Use Committee (IACUC) is: “CEEA Nord Pas de Calais” as the regional ethical commitee for animal experiments, headed by Dr Dombrowicz (President), (mail: ceea.npdc@univ-lille2.fr). It has approved the study with the acceptance number CEEA/AF 019/2009.

All animal experiments were performed following the guidelines of the Institut Pasteur de Lille animal study board, which conforms to the Amsterdam Protocol on animal protection and welfare, and Directive 86/609/EEC on the Protection of Animals Used for Experimental and Other Scientific Purposes, updated in the Council of Europe's Appendix A complied with the French law (n° 87-848 dated 19-10-1987) and the European Communities: Amendment of Cruelty to Animals Act 1976. All manipulations involving animals were carried out by qualified personnel. The animal house was placed under the direct control of the Institut Pasteur de Lille director who is the “designated responsible person” under French law. The study has been approved by Ethical Committee for experiments on animals of the Région Nord-Pas-de-Calais (ceea.npdc@univ-lille2.fr), approval number AF 19/2009.

BALB/c mice (female, 8- week-old females) were obtained from Charles River (St Germain sur l’Arbresle, France). A standardized murine TNBS colitis model was used to induce acute levels of inflammation [42]. Briefly, anesthetized mice received an intra-rectal administration of 50 ml solution of 2,4,6-trinitrobenzene sulfonic acid (TNBS, Sigma-Aldrich Chemical, France) (100 mg/kg) dissolved in 0.9% NaCl/ethanol (50/50 v/v). Following the onset of colitis, animals were observed and weighted daily according to the animal welfare standards. No excessive inflammation was depicted during this study, as observed and measured by limit points, i.e. i) body weight loss over 20% of initial ii) prominent haunchbacked posture and iii) ruffled fur. Mice were euthanized by prompt cervical dislocation performed by trained individuals. Colons were removed at sacrifice, 72 h after administration, washed and opened. Inflammation grading was performed blindly using the Wallace scoring method [43], reflecting both the intensity of inflammation and the extent of the lesions. The protective effect of LAB was studied following 5-day s-intragastric administration of 10^8^ CFU live bacteria in physiologic buffer or vehicle. A commercial preparation of prednisone (Cortancyl, Sanofi Aventis, France) was used as positive control of protection and was also orally administered for 5 subsequent days at 10 mg/kg. Histological analysis was performed on May-Grünwald-Giemsa stained 5 µm tissue sections from colon samples fixed in 10% formalin and embedded in paraffin and tissue lesions were scored according to the Ameho criteria [44].

### Statistical Analysis

Data are expressed as the mean+/−standard deviation. Statistical analysis was determined using unpaired Student *t* test and One-Way ANOVA using GraphPad Prism Sofware version 5.00 Demo (San Diego, CA). Differences were considered statistically significant if p<0.05. Survival curves were compared using the log rank survival significance test, provided by GraphPad Prism 4 statistical software package.

Epithelial co-cultures were performed 3 times independently (n = 3). For each set of experiments, an internal control of HT-29 cells stimulated by pro-inflammatory cytokines without bacteria was used. Results were expressed in comparison to this internal control.

For heterotypic co-cultures data were expressed as the mean+/−standard deviation of triplicates. Experiments were performed with two different donors. Statistical analysis was determined using unpaired Student t test and One-Way ANOVA using GraphPad Prism Sofware version 5.00 Demo (San Diego, CA). Differences were considered statistically significant if p<0.05.

For *in vivo* colitis experiments, comparisons between the different animal groups were analyzed by the non-parametric one–way analysis of variance, Mann-Whitney U test. Differences were considered to be statistically significant when the p-value was <0.05.

## Results

### Antioxidant Screening and *C. elegans* Lifespan

**Figure 1:**
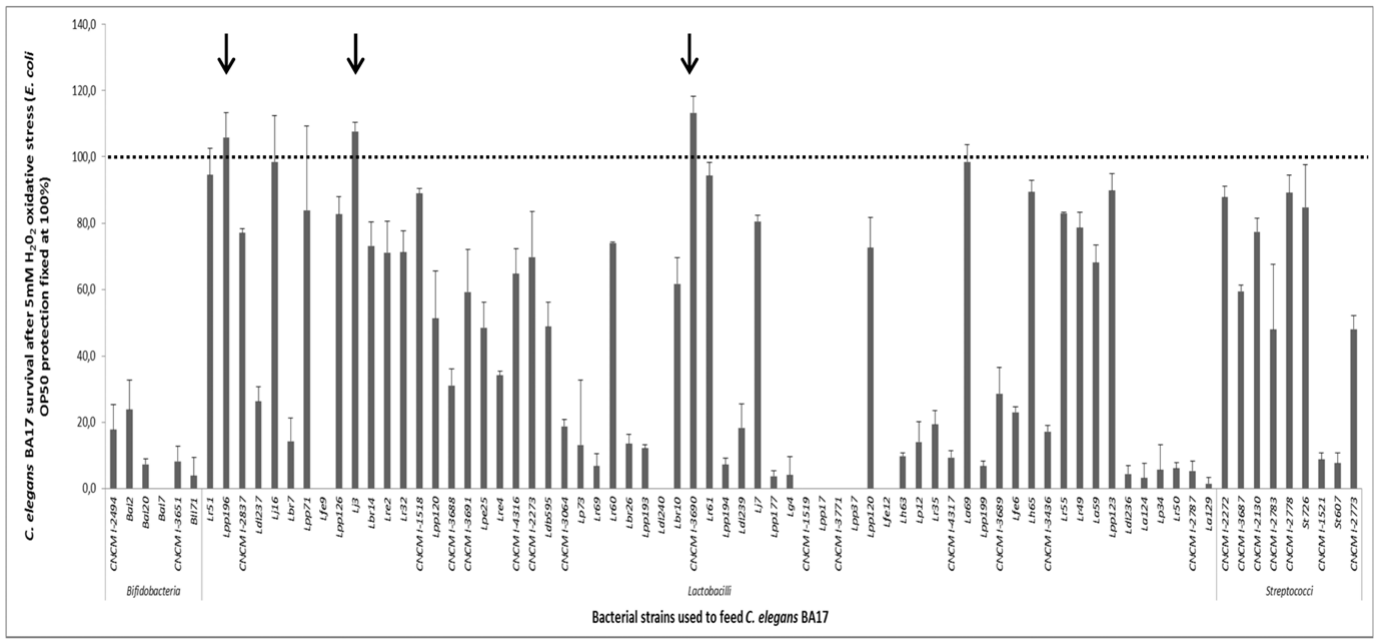
Screening for antioxidant bacteria in *C. elegans*. Survival upon a 5 mM H_2_O_2_ treatment for 5 h of *C. elegans* BA 17 strain fed for 3 days with *Bifidobacteria, Lactobacilli* and *Streptococci* strains in liquid medium. Worms were fed with *E. coli* OP 50, for larvae 1^st^ stage synchronization. E. coli OP 50 strain was inhibited with antibiotics prior of LAB and *Bifidobacteria* feeding. Protection by *E. coli* OP50 is fixed at 100%.

A pre-selection was made of 78 strains of lactic acid bacteria (LAB) and Bifidobacteria, taking into account their resistance to *in vitro* assays mimicking gastro-intestinal stresses, food safety and growth properties in acidic conditions. As a preliminary step, we verified that *C. elegans* exhibits similar growth on LAB strains as on *E. coli* OP50 strains, routinely used for feeding. Next, we fed worms with each of the 78 bacterial strains and we tested *C. elegans* survival rates upon H_2_O_2_-induced stress. Survival upon oxidative stress was determined in *C. elegans* BA17 by measuring worm viability after a 5-hour-long treatment at the previously determined optimal dose of 5 mM H_2_O_2_ [12]. To better determine LAB strain-induced protective effects, we compared them to the protective effect exerted by the control *E. coli* OP50, set at 100% (Figure 1). Under liquid experimental conditions and without any oxidative stress, LAB and Bifidobacteria didn’t affect worm viability (Figure S1). Upon oxidative stress, *Bifidobacteria* and *Streptococci* strains did not confer resistance to oxidative stress and only very few strains of *Lactobacillus* (strains Lpp196, Lj3 and CNCM I-3690, corresponding to *L. casei*, *L. jonhsonii* and *L.* *rhamnosus*, respectively) were able to confer a slight resistance to oxidative stress at a higher level than that conferred by the control strain (more than 100%). To validate the previous results, a second antioxidant screening was performed, in which the three potentially antioxidant strains identified in the first analysis were fed to *C. elegans* wild type N2 on agar plates. In this assay, the viability of worms was determined after being subjected to a treatment with 3 mM of H_2_O_2_ for 5 h. In these solid medium conditions, only the *L. rhamnosus* CNCM I-3690 proved significantly effective against oxidative stress with a level of protection of 171.3% (+/−8.1%) versus 67.6% (+/−2.1%) for Lpp196. The strain Lj3 didn’t show any protection in agar plates.

**Figure 2:**
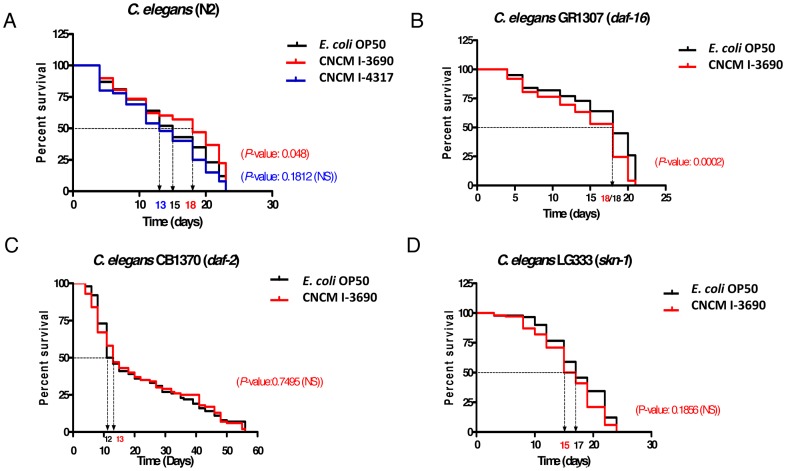
*Lactobacillus rhamnosus* CNCM I-3690 increases lifespan of *C. elegans* wild-type N2 strain and this effect is at least partially dependent of the DAF2-DAF16 pathway. A. *C. elegans* N2 wild-type strain was fed with *L. rhamnosus* CNCM I-3690, CNCM I-4317 and *E. coli* OP 50 and survival of worms was followed for 21 days. Mean lifespan, indicating the time in days where half of the worm population is still alive, is shown on the X-axis for the three situations. Curves comparisons are indicated (P-values) CNCM I-3690/CNCM I-4317 vs *E. coli* OP50 (red) and CNCM I-4317 vs *E. coli* OP50 (blue). NS: Non statistical difference. B, C, D. *C. elegans daf-16* (GR1307), *daf-2* (CB1370) and *skn-1* (LG333) loss-of-function mutant strains were fed with *L.* *rhamnosus* CNCM I-3690 and *E. coli* OP 50. Survival of worms was followed up for 21 days. B. Curves comparisons vs *E. coli* OP50 are indicated in red (P-values).

To further confirm the antioxidant properties of *L. rhamnosus* CNCM I-3690, and to analyze its potential anti-aging effect, we fed *C. elegans* wild type N2 with this strain and compared the lifespan of these worms with those fed on *E. coli* OP50. Figure 2A shows that feeding with strain CNCM I-3690 improved the average lifespan with 3 days compared to worms fed with the OP50 (lifespan of 18 *versus* 15 days). The protective effect of *L. rhamnosus* CNCM I-3690 became evident in worms after 7 days of feeding and a 20% increase in worm survival was recorded after 15 days.

Altogether, these results show that, among the 78 LAB strains analyzed; only *Lactobacillus rhamnosus* CNCM I-3690 strain induced a strong resistance to oxidative stress in *C. elegans*.

### Comparative Transcriptomic Analysis in *C. elegans* Reveals a Specific Profile for CNCM I-3690 Strain

To better understand the mechanisms underlying the antioxidant effect and longevity in *C. elegans* induced by CNCM I-3690 consumption, we performed a comparative transcriptomic analysis. To do so, transcriptomes of CNCM I-3690 were compared to those of a control *L. rhamnosus* strain, CNCM I-4317, unable to reverse oxidative stress in the previous screening (9.3% of protection, see Figure 1), or to produce a statistically significant increase in *C. elegans* lifespan (Figure 2A and Table S2). Worms were fed on either *L. rhamnosus* strains (CNCM I-3690 or CNCM I-4317) or on *E. coli* (OP50) for 3 days and antioxidant effects were monitored. It confirmed that *L. rhamnosus* CNCM I-3690 was the only strain with an antioxidative phenotype in worms in agar plates (data not shown).

A comparative transcriptomic analysis of the three strains was carried out using the young developmental stage. In young *C.* *elegans* fed on CNCM I-3690, microarrays revealed 25 differentially expressed genes compared to CNCM I-4317-fed worms, and 1278 differentially expressed genes compared to OP50-fed worms (see Supplementary Material S2 for a detailed table of these genes). By comparing the two set of differentially expressed genes, we found 18 of the 25 genes differentially expressed in worms fed with CNCM I-3690 vs CNCM I-4317 also differentially expressed in worms fed with CNCM I-3690 vs *E. coli* OP50 (see Supplementary Material S3 for the details of these 18 genes). On the other hand, 7 genes were not found among the 1278 genes. (The details of these 7 genes are presented in Supplementary Material S4). Focusing our attention on the differences between the two *L. rhamnosus* strains, two genes were down-regulated and 23 genes were up-regulated (Table 1). Most of the up-regulated genes in young worms are involved in the insulin-like pathway, lipid metabolism and stress response. Conversely, in adult worms (fed with the strains for 10 days) fewer genes were differentially expressed for the three treatments, being 4, 113 and 196 for CNCM I-3690 *versus* CNCM I-4317, CNCM I-3690 *versus* OP50 and CNCM I-4317 *versus* OP50, respectively (see Supplemental Material S5, S6 and S7 for the details of these 4, 133 and 196 genes). Interestingly, transcriptional factor DAF-16 is involved in all three pathways modulated upon ingestion of *L. rhamnosus* CNCM I-3690. Moreover, downstream DAF-16 up-regulation could also explain the observed increase in lifespan after ingestion of strain CNCM I-3690 (see above). Finally, we confirmed the microarray data by RT-qPCR for the *arf-1.1* and *gst-22* genes (see Supplementary Material S8 for experimental details and results).

**Table 1:** Transcriptomic analysis of *C. elegans* N2 fed with CNCM I-3690 vs CNCM I-4317.

Up regulated genes							
Gene Symbol	Description	Fold change		p-Value			adjusted p-Value
lec-11	gaLECtin	2,7		1,27E–06			0,02866
ZK593.3	hypothetical protein	2,4		4,57E–06			0,05164
F23F12.12	hypothetical protein	7,7		1,66E–06			0,05462
cpr-3	hypothetical protein	2,3		3,07E–05			0,05462
Y71H2AM.16	hypothetical protein	2,3		2,61E–05			0,05462
spp-16	SaPosin-like Protein family	1,8		3,13E–05			0,05462
R08E5.3	hypothetical protein	2,7		2,39E–05			0,05462
C06A12.3	hypothetical protein	1,6		1,94E–05			0,05462
clc-1	CLaudin-like protein	2,4		2,59E–05			0,05462
gst-22	Glutathione S-Transferase	3,4		3,14E–05			0,05462
fat-7	FATty acid desaturase	4,4		1,66E–05			0,05462
T04F3.1	hypothetical protein	2,2		2,62E–05			0,05462
F44E7.5	hypothetical protein	1,8		4,48E–05			0,07109
acl-12	ACyLtransferase-like	2,5		6,88E–05			0,07786
Y34F4.2	hypothetical protein	2,1		6,20E–05			0,07786
ric-3	hypothetical protein	1,4		6,71E–05			0,07786
hsp-12.3	Heat Shock Protein	1,9		6,24E–05			0,07786
Peroxidase	hypothetical protein	1,7		7,33E–05			0,07897
F49E2.5	hypothetical protein	2,3		7,78E–05			0,08005
pqn-60	Prion-like-(Q/N-rich)-domain-bearing protein	2,1		9,14E–05			0,08833
D2092.1	hypothetical protein	1,6		9,37E–05			0,08833
W03D8.8	hypothetical protein	2,4		0,00011			0,09700
cpr-3	Cysteine Protease related	2,5		0,00011			0,09816
arf-1.1	ADP-Ribosylation Factor related	3,6		0,00012			0,09816
**Down regulated genes**							
**Gene Symbol**	**Description**		**Fold change**		**p-Value**	**adjusted p-Value**	
T10B10.3	hypothetical protein		−1,8		1,58E-05	0,05462	
F33A8.6	hypothetical protein		−1,6		4,71E-05	0,07109	

### *L. rhamnosus* CNCM I-3690 Modulates DAF2/DAF-16 in *C. elegans*

To elucidate the role of DAF-16, we studied the antioxidant activity and effects on lifespan of *L. rhamnosus* CNCM I-3690 in *C. elegans daf-2* (CB1370), *daf-16* (GR1307) and *skn-1* (LG333) mutant backgrounds. Unfortunately, both H_2_O_2_ doses of 5 mM and 3 mM proved toxic for these mutant strains and therefore the anti-oxidative phenotype could not be evaluated. Notwithstanding, effects on lifespan could be assessed. Data indicated that the increase in survival after 15 days observed in wild-type strain N2 was absent in GR1307, CB1370 and LG333 mutant worms; furthermore, there was no statistical difference in mean lifespan after consumption of CNCM I-3690 or OP50 bacterial strains (Figure 2B, C and D and Table S2). This would indicate that the increased lifespan observed after CNCM I-3690 consumption in wild-type N2 is dependent (at least partially) on the DAF-2/DAF-16 signaling pathway.

**Figure 3:**
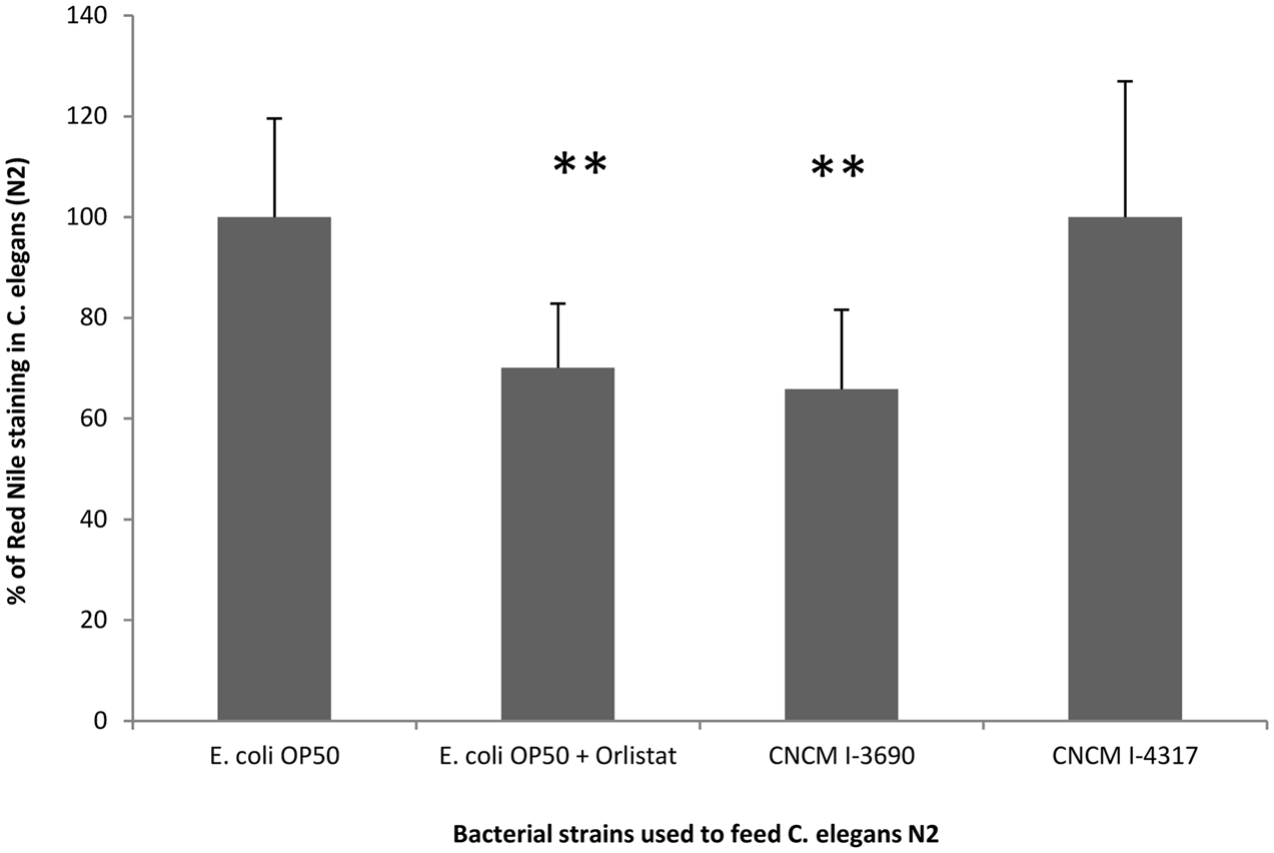
*Lactobacillus rhamnosus* CNCM I-3690 inhibits total fat deposit in *C. elegans* wild-type N2. Fluorescence of Red Nile stained *C. elegans* wild-type N2 strain fed with *L. rhamnosus* CNCM I-3690, CNCM I-4317, *E. coli* OP50 and *E. coli* OP50 with orlistat as a positive control. **p*-value*≤0,001. Fat deposit was measured by fluorescence counting.

Long-lived worms are reported to display increased fat accumulation and altered metabolism [45]. Adult loss-of-function mutants in *daf-2*, corresponding to the *C. elegans* insulin-receptor, accumulate fat; indeed, DAF-16 transcriptional factor has been shown to control fat storage-related pathways [45]. For this reason, we studied lipid inclusion in worms fed on *L. rhamnosus* CNCM I-3690 and CNCM I-4317, and on *E. coli* OP50. We observed that worms fed with strain CNCM I-3690 showed a reduction of 34% in lipid inclusion when compared with worms fed on OP50 or CNCM I-4317 (Figure 3). Remarkably, this phenotype is partially dependent on *fat-7* gene encoding delta-9 fatty acid desaturation enzyme, since in a *fat-7* mutant (BX153) the decrease in lipid inclusion was reduced to 9% vs 34% in wild-type N2 (p-value<0.001). Interestingly, *fat-7* was one of the lipid metabolism pathways up-regulated by *L. rhamnosus* CNCM I-3690 according to the transcriptomic analysis (Table 1).

In conclusion, transcriptome data indicate that the *L. rhamnosus* CNCM I-3690 strain diferentially modulates the DAF-16 dependent insulin-like pathway in *C. elegans*. The transcriptional activation of the DAF-16 pathway by the strain is in agreement with its phenotype of improved resistance to H_2_O_2_, its longevity and decreased lipid inclusion. Interestingly, these phenotypes are all associated with a reduction of inflammatory processes. In addition there is evidence in mammalian systems for a direct link between signaling via longevity factors, such as FoxOs or SIRT-1, and inhibition of NF-κB signaling [46], [47]. Thus, we hypothesized that a strain providing antioxidant protection in the *C. elegans* model through the DAF-16 transcriptional factor may also exhibit an anti-inflammatory profile in mammals. Therefore we decided to focus further experimentation on the analysis of anti-inflammatory properties of both strains CNCM I-3690 and the control CNCM I-4317 in *in vitro* and *in vivo* models.

### *In vitro* Anti-inflammatory Profile of *L. rhamnosus* CNCM I-3690

**Figure 4:**
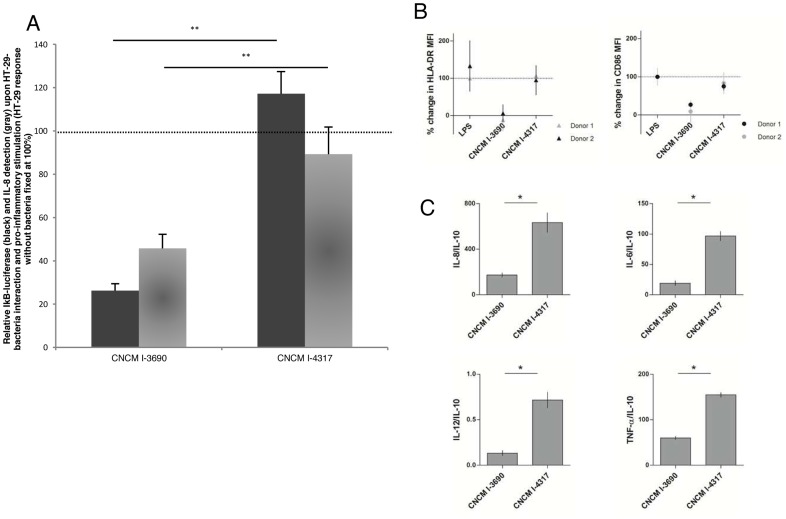
*Lactobacillus rhamnosus* CNCM I-3690 has an anti-inflammatory effect *in vitro*. A. Relative IL-8, production and I-κB-luciferase detection from the HT-29-bacteria interaction assays. Each bar represent the mean value of three replicate samples and and error bars depict corresponding standard deviation. Black bars: I-kB-luciferase, grey bars: IL-8. **p-value ≤0.05. B. Phenotypical analysis of monocyte-derived DCs co-cultured with HT-29-NF-κB-luciferase cells. Cells were incubated with LPS or LPS+bacteria. CNCM I-3690 down-regulated the expression of HLA-DR and CD86 surface markers. Results were expressed according to the following equation [(bacteria+LPS)-LPS]/LPS-basal*100. Experiments were performed with two different donors. C. Cytokine ratios (IL-8/IL-10, IL-6/IL-10, IL-12/IL-10 and TNF-α/IL-10) of DCs from donor 2 in co-culture with HT-29-NF-κB-luciferase cells and bacteria w or w/o LPS. Experiments were performed with two different donors (data shown for donor 2). *p-value≤0.05.

To validate the potential anti-inflammatory phenotype of the *L.* *rhamnosus* CNCM I-3690 strain in mammals, we studied the effect of a transient co-culture of CNCM I-3690 and CNCM I-4317 with HT-29 intestinal epithelial cell-line, after 6 h of pro-inflammatory stimulation with 50 ng TNF-α, 2.5 ng IL-1β and 7.5 ng IFN-γ. We observed that a two-hour pre-incubation of epithelial cells with *L. rhamnosus* CNCM I-3690 induced a significant reduction in NF-κB signaling observed by an I-κB reporter system and IL-8 after 6 h of pro-inflammatory stimulation when compared with CNCM I-4317 (Figure 4A). We also studied the effect of co-culturing *L. rhamnosus* CNCM I-3690 and CNCM I-4317 in a transwell-filter co-culture system with HT-29-NF-κB-luciferase epithelial cells in the apical side and human dendritic cells (DCs), obtained from human blood donors, in the basal side. A clear anti-inflammatory profile was induced by strain CNCM I-3690 in comparison with CNCM I-4317 (Figures 4B and 4C). In fact, phenotype of DCs was different when co-cultured with strains CNCM I-3690 or CNCM I-4317 in direct contact with epithelial cells. When the system was co-cultured with CNCM I-3690, we observed a 90% reduction in CD86 and HLA expression, which was similar to the LPS control for the co-culture with CNCM I-4317 (Figure 4B). We also observed that only CNCM I-3690 was able to reduce the pro-inflammatory cytokine ratios IL-12/IL-10, IL-6/IL-10, IL-8/IL-10 and TNFα/IL-10 measured in the co-culture system (Figure 4C). Moreover, we confirmed the reduction in NF-κB signaling in the context of the co-culture system with DCs, as previously shown with a single bacteria/epithelial cell interaction.

### *L. rhamnosus* CNCM I-3690 Alleviates Colitis Symptoms in a TNBS-induced Recto-colitis Murine Model

**Figure 5:**
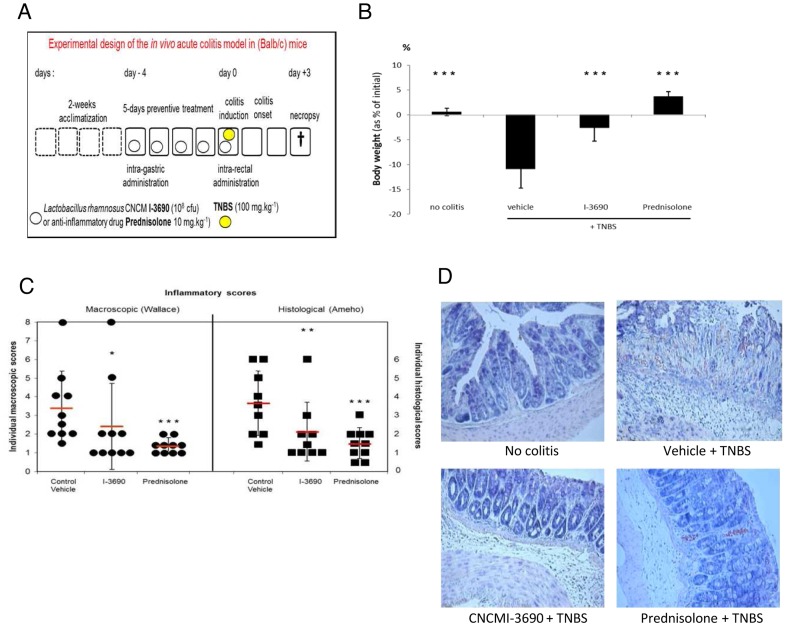
Protective effect of *Lactobacillus rhamnosus* CNCM I-3690 in a TNBS-induced murine model of colitis. A. Design of the interventional animal study. B. 3-day post colitis body-weight losses (% of initial) for healthy control mice, vehicle-TNBS-treated animal and either *L. rhamnosus* CNCM I-3690-fed TNBS-treated mice or Prednisolone-TNBS-treated mice. Data represent the mean+/−SEM, (number of mice n = 10); ***: P<0.001. C: Individual inflammatory macroscopic (Wallace score, left panel), and histological damage scores (Ameho score, right panel) in Control, probiotic and drug treatment conditions respectively, means +/− SD are indicated, **: P<0.01, ***: P<0.001. D: Representative histological colon sections from mice treated in various conditions after induction of TNBS colitis; May-Grünwald and Giemsa-stainings of 5 µm paraffin sections, original magnification×40.

As the anti-oxidative and anti-inflammatory potential of *L. rhamnosus* CNCM I-3690 was confirmed by both the *C. elegans* model and the *in vitro* eukaryotic cell co-culture experiments, we went on to analyze the effects of this strain in a mouse model of intestinal inflammation. Accordingly, the effect of intra-gastric administration of 10^8^ CFU/mL of strain CNCM I-3690 was evaluated over a 5-day period in an acute TNBS-induced recto-colitis gold-standard model, and compared with administration of an anti-inflammatory drug (Figure 5A). In comparison with the vehicle-TNBS-treated group, the 3-day post colitis body-weight losses were significantly lower when mice were fed strain CNCM I-3690 (respectively 11% +/−3.7 and 2.6% +/−3.7, p<0.001), Figure 5B. Similarly, there was nearly a 30% reduction in macroscopic lesions, as recorded by the mean Wallace scores, in the group that received strain CNCM I-3690, compared to the placebo (non-LAB) control group (from 3.4+/−0.8 to 2.4+/−0.77, p<0.01). Notably, the anti-inflammatory effect of strain CNCM I-3690 was similar to the prednisolone positive control, which showed a mean score of 1.5+/−0.13 and provided 58% of protection (p<0.001) (Figure 5C). Histological features confirmed *L. rhamnosus* CNCM I-3690 ability to prevent inflammation in this model (Figure 5C and D), the latter showing considerably fewer inflammatory cell infiltrates (neutrophils) and mucosal and submucosal lesions.

Taken together, these data confirm that, in the *C. elegans* model, the antioxidant effect exerted by this strain, selected from among 78 potential candidates, is associated with the anti-inflammatory profile, which was observed in host-bacteria interactions *in vitro* and in pre-clinical assays of TNBS-induced colitis in mice. These results clearly demonstrate that *C. elegans* is an efficient model for screening strains for potential probiotic properties and suggest that *L. rhamnosus* CNCM I-3690 is a novel anti-inflammatory potential probiotic strain.

## Discussion

*Caenorhabditis elegans* was previously shown to be a useful tool for probiotic screening and identification. The potential antitumor activity and the growth inhibition of *Lactobacillus salivarius*, *Lactobacillus reuteri* and *Pediococcus acidilactici* were reported [48]. Some probiotic characteristics of a newly isolated *Lactobacillus spp*. were studied in terms of their impact on *C. elegans* longevity [49], while *C. elegans* was also used to preselect *Lactobacillus* isolates potentially able to control *Salmonella typhimurium* infection [50]. Our study describes an original approach, which uses the nematode *C. elegans* as a preclinical model not only to screen potential antioxidant probiotic strains, but also to identify the underlying molecular mechanisms of action triggered by the selected strains in the host. Interestingly, most of the pathways involved in protection against oxidative stress are highly conserved in higher organisms. Indeed, *C. elegans* and human genomes have *ca* 35% homology [51] and it is noteworthy that the number of human disease-related genes sharing at least modest homology with *C. elegans* can range from 40–75% [52]. Moreover, components of insulin signaling pathways have been conserved, such as kinases, nuclear hormone receptors, F-box proteins and transcription factors [53]. Here, we exploit the technical advantages of the nematode model to obtain new mechanistic insights on potential probiotic strains and to avoid the excessive use of mammals. Similar approaches have been used to identify conserved mechanisms in neuronal disorders [54] and to screen new antimicrobial or antifungal compounds *in vivo* upon infection of *C. elegans* and study its innate immune response [55], [56], [57].

Using the strategy described above, a *Lactobacillus rhamnosus* strain, CNCM I-3690, was identified among 78 bacterial strains as providing protection and increasing longevity in *C. elegans*. The transcriptome analysis showed that the consumption of this strain by worms activates genes downstream of the DAF-16/FOXO pathway, controlling the insulin-like pathway, lipid metabolism in *C. elegans* and increasing mean lifespan of worms. These results suggest that CNCM I-3690 strain has a health-aging effect in worms which needs to be confirmed in higher organisms. Transcriptomic analysis also showed that worms fed on CNCM I-3690 for 3 days, over-expressed the fatty acid Δ9 stearoyl-CoA desaturase FAT-7. In mammals, desaturases play a key role in maintaining appropriate fatty acid desaturation levels, which are important for membrane functioning and lipid metabolism control. In *C. elegans*, FAT-7, FAT-6 and FAT-2 desaturases also catalyze the first step in the synthesis of Poly-Unsaturated Fatty Acids (PUFAs) [45]. Several studies show that FAT-7 expression is controlled by “Nuclear Hormone Receptors” (NHRs), especially NHR-8, NHR-49 and NHR-80, which in turn controls lipid metabolism and nematode lifespan [58]–[59], [60]. Interestingly, it was shown that PUFAs can act as regulators of the insulin-like IGF-II pathway, and DAF-16, in particular, could be one of the transcriptional factors involved in fatty acid homeostasis in *C. elegans* by controlling the expression of FAT-2, FAT-6 and FAT-7 desaturases and elongases [61]. In this work, we observed a FAT-7 dependent lipid inclusion in worms fed with I-3690 alone, which supports the hypothesis that this strain directly mediates DAF-16-controlled desaturase regulation and lipid metabolism. The mechanisms whereby this strain acts directly on the worm lipid homeostasis remain unknown; notwithstanding, a possible correlation between these results and the increased lifespan of worms fed on CNCM I-3690 cannot be ruled out.

Although further *in vivo* corroboration is required, these results suggest that some *L. rhamnosus* CNCM I-3690 ligands could act direct or indirectly on the DAF-16/FOXO and/or SKN-1 pathways through the lumen of *C. elegans* intestine, by a mechanism as yet unknown. One may hypothesize that some bacterial ligands could trigger the DAF-16 transcription factor by its de-phosphorylation, either by the upstream AKT kinase cascade or by another mechanism that allows DAF-16 to translocate to the nucleus [62]. *L. rhamnosus* CNCM I-3690 has a strong anti-inflammatory profile in co-culture with intestinal epithelial cell-lines, *in vitro* and this was confirmed in a TNBS-induced colitis model in mice.

Interestingly, similar effects have been observed in other *L. rhamnosus* strains. In this respect, *L. rhamnosus* GR-1 suppresses TNFα signaling in macrophages through a G-CSF-mediated inhibition of JNK [63]. Furthermore, recent findings have shown that *Lactobacillus rhamnosus* GG generates Extracellular Related Kinase (ERK)-mediated cellular ROS through formyl peptide receptors and modulation of MAP kinase phosphatase redox status [64], [65].

These results correlate the antioxidant effects of *L. rhamnosus* CNCM I-3690 in *C. elegans* with the strong anti-inflammatory profile of this strain when co-cultured with human epithelial and/or Dendritic Cells and, likewise, its ability to reduce inflammation in a murine model of colitis. New *in vivo* assays are currently underway to shed light on the pathways involved in the anti-inflammatory effect in mice.

In summary, this study shows that *C. elegans* is a promising pre-clinical model for screening potential probiotic strains in a faster and simpler way than other more classical approaches. This simple model enables us to study potential probiotic effects based on simple read-outs, such as oxidative stress, and then correlate these effects with more complex models *in vitro* or *in vivo*. Further studies must be performed to better characterize the potential probiotic effects identified here.

## Supporting Information

Figure S1 ***C. elegans*** **BA17 can be fed with** ***Bifidobacteria*** **,** ***Lactobacilli*** **and** ***Streptococci*** **strains.** Survival of *C. elegans* BA 17 strain fed for 3 days with *Bifidobacteria*, *Lactobacilli* and *Streptococci* strains in liquid medium was followed, after larvae 1st stage synchronization with *E. coli* OP 50 and without any oxidative stress. Protection by *E. coli* OP50 is fixed at 100%.
(TIF)

Table S1 **Genera and species specifications for all the bacterial strains used in this work.** Genera, species and subspecies are specified for each bacterial strain used in this study, with their corresponding code names.
(DOCX)

Table S2 **DAF-16, DAF-2 and SKN-1 play an essential role in** ***C. elegans*** **longevity resulting from feeding with** ***Lactobacillus rhamnosus*** **CNCM I-3690.** See corresponding data in Figure 2. *NS: No significant differences between control conditions (NGM+*E. coli* OP50) and treatment conditions (NGM + CNCM I-3690). Statistical analysis was performed with GraphPad prism 4 using Log Rank Test.
(DOCX)

Supplementary Material S1 **LAB and bifidobacteria growth curves.** Growth curves of representative strains for each genus, *Streptococcus* CNCM I-2778, *Lactobacillus* CNCM I-3064 and *Bifidobacterium* Bal7, and detailed protocols are presented.
(DOCX)

Supplementary Material S2 **List of the 1278 differentially expressed genes in** ***C. elegans*** **fed with** ***L. rhamnosus*** **CNCM I-3690 versus** ***E. coli*** **OP50.** *C. elegans* N2 wild-type strain was fed with *L. rhamnosus* strain CNCM I-3690 and *E. coli* strain OP50 during 3 days. Transcriptome profiling was performed comparing worms fed with the two bacterial strains. RNA samples were hybridized (in triplicate) onto *C. elegans* Genome Array (Affymetrix), for details see Materials. Genes were considered differentially expressed when the multiple testing adjusted P-value<0,01.
(XLS)

Supplementary Material S3 **List of the 18 genes differentially expressed in** ***C. elegans*** **fed with** ***L. rhamnosus*** **CNCM I-3690 versus** ***L. rhamnosus*** **CNCM I-4317 and in** ***C. elegans*** **fed with** ***L. rhamnosus*** **CNCM I-3690 versus** ***E. coli*** **OP50.** *C. elegans* N2 wild-type strain was fed with the three strains *L. rhamnosus* CNCM I-3690, *L. rhamnosus* CNCM I-4317 and *E. coli* OP50 during 3 days. Transcriptome profiling was performed comparing worms fed with the three bacterial strains. RNA samples were hybridized (in triplicate) onto *C. elegans* Genome Array (Affymetrix), for details see Materials. Genes were considered differentially expressed when the multiple testing adjusted P-value<0,01.
(XLS)

Supplementary Material S4 **List of the 7 differentially expressed genes not shared between** ***C. elegans*** **fed with** ***L. rhamnosus*** **CNCM I-3690 versus** ***L. rhamnosus*** **CNCM I-4317 and in** ***C. elegans*** **fed with** ***L. rhamnosus*** **CNCM I-3690 versus** ***E. coli*** **OP50.** *C. elegans* N2 wild-type strain was fed with the three strains *L. rhamnosus* CNCM I-3690, *L. rhamnosus* CNCM I-4317 and *E. coli* OP50 during 3 days. Transcriptome profiling was performed comparing worms fed with the three bacterial strains. RNA samples were hybridized (in triplicate) onto *C. elegans* Genome Array (Affymetrix), for details see Materials. Genes were considered differentially expressed when the multiple testing adjusted P-value<0,01.
(XLS)

Supplementary Material S5 **List of the 4 differentially expressed genes** ***C. elegans*** **fed for 10 days with** ***L.*** ** ***rhamnosus*** **CNCM I-3690 versus** ***L. rhamnosus*** **CNCM I-4317.** *C. elegans* N2 wild-type strain was fed with both *L. rhamnosus* strains CNCM I-3690 and CNCM I-4317 during 10 days. Transcriptome profiling was performed comparing worms fed with the two bacterial strains. RNA samples were hybridized (in triplicate) onto *C. elegans* Genome Array (Affymetrix), for details see Materials. Genes were considered differentially expressed when the multiple testing adjusted P-value<0,01.
(XLS)

Supplementary Material S6 **List of the 133 differentially expressed genes** ***C. elegans*** **fed for 10 days with** ***L. rhamnosus*** **CNCM I-3690 versus** ***E. coli*** **OP50.** *C. elegans* N2 wild-type strain was fed with *L. rhamnosus* CNCM I-3690 and *E. coli* OP50 strains during 10 days. Transcriptome profiling was performed comparing worms fed with the two bacterial strains. RNA samples were hybridized (in triplicate) onto *C. elegans* Genome Array (Affymetrix), for details see Materials. Genes were considered differentially expressed when the multiple testing adjusted P-value<0,01.
(XLS)

Supplementary Material S7 **List of the 196 differentially expressed genes** ***C. elegans*** **fed for 10 days with** ***L.*** ** ***rhamnosus*** **CNCM I-4317 versus** ***E. coli*** **OP50.** *C. elegans* N2 wild-type strain was fed with *L. rhamnosus* CNCM I-4317 and *E. coli* OP50 strains during 10 days. Transcriptome profiling was performed comparing worms fed with the two bacterial strains. RNA samples were hybridized (in triplicate) onto *C. elegans* Genome Array (Affymetrix), for details see Materials. Genes were considered differentially expressed when the multiple testing adjusted P-value<0,01.
(XLS)

Supplementary Material S8 **Confirmation of microarray data by RT-qPCR for** ***arf-1.1*** **and** ***gst-22*** **genes.** Expression ratios for *arf-1.1* and *gst-22* genes were obtained by RT-qPCR. All experiments were repeated at least three times (biological replicates) and were internally controlled (technical replicate). Expression changes were obtained by calculating the relative expression levels using the 2^−ΔΔCT^ method. See Materials and Methods for details.
(DOCX)
