# Supplementary figures and images for: Anti-Inflammatory Lactobacillus rhamnosus CNCM I-3690 Strain Protects against Oxidative Stress and Increases Lifespan in Caenorhabditis elegans
Source: PLoS One. 2012 Dec 26;7(12):e52493. doi: 10.1371/journal.pone.0052493 (PMC3530454; doi:10.1371/journal.pone.0052493)

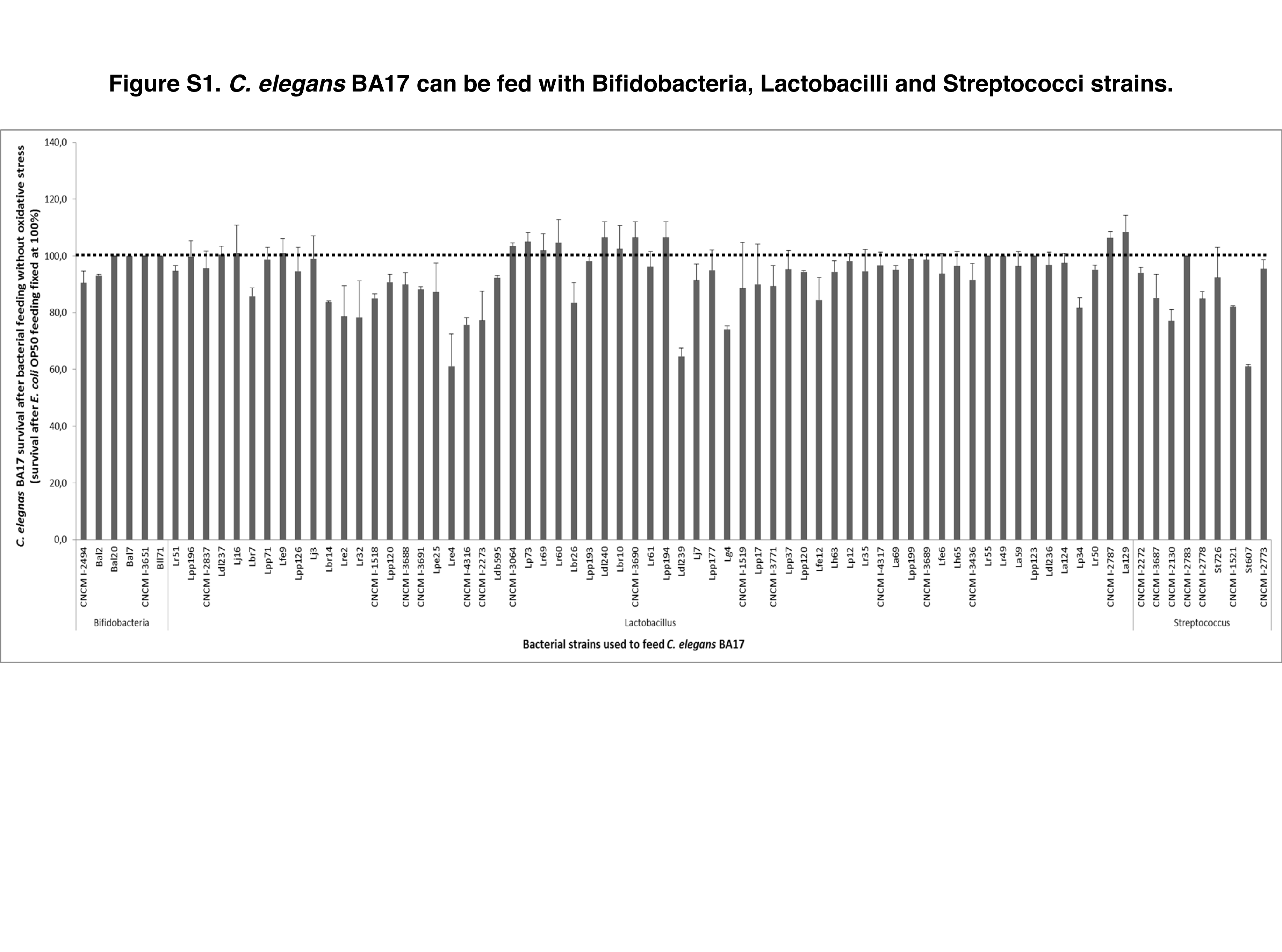

Supplement: Figure S1 — C. elegans BA17 can be fed with Bifidobacteria , Lactobacilli and Streptococci strains. Survival of C. elegans BA 17 strain fed for 3 days with Bifidobacteria, Lactobacilli and Streptococci strains in liquid medium was followed, after larvae 1st stage synchronization with E. coli OP 50 and without any oxidative stress. Protection by E. coli OP50 is fixed at 100%. (TIF) [file pone.0052493.s001.tif]
