# Supplementary material for: Anti-Inflammatory Lactobacillus rhamnosus CNCM I-3690 Strain Protects against Oxidative Stress and Increases Lifespan in Caenorhabditis elegans
Source: PLoS One. 2012 Dec 26;7(12):e52493. doi: 10.1371/journal.pone.0052493 (PMC3530454; doi:10.1371/journal.pone.0052493)
Supplement: Table S1 — Genera and species specifications for all the bacterial strains used in this work. Genera, species and subspecies are specified for each bacterial strain used in this study, with their corresponding code names. (DOCX) [file pone.0052493.s002.docx]

**Supplementary Table S1. Genera and species specifications for all the bacterial strains used in this work**

| Strain codes used | Genera and species designation for the strains used in this work |
| --- | --- |
| CNCM I-2494 | *Bifidobacterium animalis* subsp*. lactis* |
| Bal2 | *Bifidobacterium animalis* subsp*. lactis* |
| Bal20 | *Bifidobacterium animalis* subsp*. lactis* |
| Bal7 | *Bifidobacterium animalis* subsp*. lactis* |
| CNCM I-3651 | *Bifidobacterium bifidum* |
| Bll71 | *Bifidobacterium longum* subsp*. longum* |
| Lr51 | *Lactobacillus rhamnosus* |
| Lpp196 | *Lactobacillus paracasei* subsp*. paracasei* |
| CNCM I-2837 | *Lactobacillus delbrueckii* subsp*. bulgaricus* |
| Ldl237 | *Lactobacillus delbrueckii* subsp*. lactis* |
| Lj16 | *Lactobacillus johnsonii* |
| Lbr7 | *Lactobacillus breve* |
| Lpp71 | *Lactobacillus paracasei* subsp*. paracasei* |
| Lfe9 | *Lactobacillus fermentum* |
| Lpp126 | *Lactobacillus paracasei* subsp*. paracasei* |
| Lj3 | *Lactobacillus johnsonii* |
| Lbr14 | *Lactobacillus breve* |
| Lre2 | *Lactobacillus reuteri* |
| Lr32 | *Lactobacillus rhamnosus* |
| CNCM I-1518 | *Lactobacillus paracasei* subsp*. paracasei* |
| Lpp120 | *Lactobacillus paracasei* subsp*. paracasei* |
| CNCM I-3688 | *Lactobacillus delbrueckii* subsp*. lactis* |
| CNCM I-3691 | *Lactobacillus fermentum* |
| Lpe25 | *Lactobacillus pentosus* |
| Lre4 | *Lactobacillus reuteri* |
| CNCM I-4316 | *Lactobacillus rhamnosus* |
| CNCM I-2273 | *Lactobacillus acidophilus* |
| Ldb595 | *Lactobacillus delbrueckii* subsp*. bulgaricus* |
| CNCM I-3064 | *Lactobacillus plantarum* |
| Lp73 | *Lactobacillus plantarum* |
| Lr69 | *Lactobacillus rhamnosus* |
| Lr60 | *Lactobacillus rhamnosus* |
| Lbr26 | *Lactobacillus breve* |
| Lpp193 | *Lactobacillus paracasei* subsp*. paracasei* |
| Ldl240 | *Lactobacillus delbrueckii* subsp*. lactis* |
| Lbr10 | *Lactobacillus breve* |
| CNCM I-3690 | *Lactobacillus rhamnosus* |
| Lr61 | *Lactobacillus rhamnosus* |
| Lpp194 | *Lactobacillus paracasei* subsp*. paracasei* |
| Ldl239 | *Lactobacillus delbrueckii* subsp*. lactis* |
| Lj7 | *Lactobacillus johnsonii* |
| Lpp177 | *Lactobacillus paracasei* subsp*. paracasei* |
| Lg4 | *Lactobacillus gasseri* |
| CNCM I-1519 | *Lactobacillus delbrueckii* subsp*. bulgaricus* |
| Lpp17 | *Lactobacillus paracasei* subsp*. paracasei* |
| CNCM I-3771 | *Lactobacillus delbrueckii* subsp*. bulgaricus* |
| Lpp37 | *Lactobacillus paracasei* subsp*. paracasei* |
| Lpp120 | *Lactobacillus paracasei* subsp*. paracasei* |
| Lfe12 | *Lactobacillus fermentum* |
| Lh63 | *Lactobacillus helveticus* |
| Lp12 | *Lactobacillus plantarum* |
| Lr35 | *Lactobacillus rhamnosus* |
| CNCM I-4317 | *Lactobacillus rhamnosus* |
| La69 | *Lactobacillus acidophilus* |
| Lpp199 | *Lactobacillus paracasei* subsp*. paracasei* |
| CNCM I-3689 | *Lactobacillus paracasei* subsp*. paracasei* |
| Lfe6 | *Lactobacillus fermentum* |
| Lh65 | *Lactobacillus helveticus* |
| CNCM I-3436 | *Lactobacillus plantarum* |
| Lr55 | *Lactobacillus rhamnosus* |
| Lr49 | *Lactobacillus rhamnosus* |
| La59 | *Lactobacillus acidophilus* |
| Lpp123 | *Lactobacillus paracasei* subsp*. paracasei* |
| Ldl236 | *Lactobacillus delbrueckii* subsp*. lactis* |
| La124 | *Lactobacillus acidophilus* |
| Lp34 | *Lactobacillus plantarum* |
| Lr50 | *Lactobacillus rhamnosus* |
| CNCM I-2787 | *Lactobacillus delbrueckii* subsp*. bulgaricus* |
| La129 | *Lactobacillus acidophilus* |
| CNCM I-2272 | *Streptococcus thermophilus* |
| CNCM I-3687 | *Streptococcus thermophilus* |
| CNCM I-2130 | *Streptococcus thermophilus* |
| CNCM I-2783 | *Streptococcus thermophilus* |
| CNCM I-2778 | *Streptococcus thermophilus* |
| St726 | *Streptococcus thermophilus* |
| CNCM I-1521 | *Streptococcus thermophilus* |
| St607 | *Streptococcus thermophilus* |
