# Supplementary material for: Anti-Inflammatory Lactobacillus rhamnosus CNCM I-3690 Strain Protects against Oxidative Stress and Increases Lifespan in Caenorhabditis elegans
Source: PLoS One. 2012 Dec 26;7(12):e52493. doi: 10.1371/journal.pone.0052493 (PMC3530454; doi:10.1371/journal.pone.0052493)
Supplement: Table S2 — DAF-16, DAF-2 and SKN-1 play an essential role in C. elegans longevity resulting from feeding with Lactobacillus rhamnosus CNCM I-3690. See corresponding data in Figure 2. *NS: No significant differences between control conditions (NGM+E. coli OP50) and treatment conditions (NGM + CNCM I-3690). Statistical analysis was performed with GraphPad prism 4 using Log Rank Test. (DOCX) [file pone.0052493.s003.docx]

**Supplementary Table S2. DAF-16, DAF-2 and SKN-1 play an essential role in *C. elegans* longevity resulting from feeding with CNCM I-3690**

| **Strain** | **Genotype** | **Treatment** | **Mid-Lifespan (days)** | **Log Rank X2** | **p-value** |
| --- | --- | --- | --- | --- | --- |
| **N2** | Wild-type | NGM+*E. coli* OP50 | 15 |  |  |
| **N2** | Wild-type | NGM+CNCM I-3690 | 18 | 3.911 | 0.048 |
| **N2** | Wild-type | NGM+CNCM I-4317 | 13 | 1.788 | 0.1812 (NS)* |
| **GR1307** | daf-16 | NGM+*E. coli* OP50 | 18 |  |  |
| **GR1307** | daf-16 | NGM+CNCM I-3690 | 18 | 13.82 | 0.0002 |
| **CB1370** | daf-2 | NGM+*E. coli* OP50 | 12 |  |  |
| **CB1370** | daf-2 | NGM+CNCM I-3690 | 13 | 0.101 | 0.7495 (NS)* |
| **LG333** | skn-1 | NGM+*E. coli* OP50 | 17 |  |  |
| **LG333** | skn-1 | NGM+CNCM I-3690 | 15 | 1.753 | 0.1856(NS)* |
