# Supplementary material for: Anti-Inflammatory Lactobacillus rhamnosus CNCM I-3690 Strain Protects against Oxidative Stress and Increases Lifespan in Caenorhabditis elegans
Source: PLoS One. 2012 Dec 26;7(12):e52493. doi: 10.1371/journal.pone.0052493 (PMC3530454; doi:10.1371/journal.pone.0052493)
Supplement: Supplementary Material S1 — LAB and bifidobacteria growth curves. Growth curves of representative strains for each genus, Streptococcus CNCM I-2778, Lactobacillus CNCM I-3064 and Bifidobacterium Bal7, and detailed protocols are presented. (DOCX) [file pone.0052493.s004.docx]

***Streptococcus***

***Lactobacillus***

***Bifidobacterium***

The strains belonging to *Streptococcus*, *Bifidobacterium* and *Lactobacillus* genera have being grown in Elliker, MRS with cysteine and MRS media respectively. As the bioassay of the *in vivo* antioxidant activity must be carried out with samples of living cells, cells were recovered in the log phase growth. After the analysis of the growth curves of some representative strains, we have established that cells will be recovered after 15 h of at OD^600^= 1, 1.5 and 1.7 for *Streptococcus,* *Lactobacillus* and *Bifidobacterium* respectively. The cultures of the different lactic acid bacteria obtained were added to the liquid media at a final concentration 2x10^6^ cells/mL.

For liquid media oxidative stress assays in *C. elegans*, curves were obtained using a representative strain of each genus (see above). We selected the cultures within the end of log phase to be sure that cells were taken before stationary phase starts and therefore they were alive. Then we grew the strains for 15 h. In the case of assays in agar plates, cells were pre-seeded on the agar surface and incubated 15h, so the nematodes were fed with fresh cells.
