# Supplementary material for: Anti-Inflammatory Lactobacillus rhamnosus CNCM I-3690 Strain Protects against Oxidative Stress and Increases Lifespan in Caenorhabditis elegans
Source: PLoS One. 2012 Dec 26;7(12):e52493. doi: 10.1371/journal.pone.0052493 (PMC3530454; doi:10.1371/journal.pone.0052493)
Supplement: Supplementary Material S8 — Confirmation of microarray data by RT-qPCR for arf-1.1 and gst-22 genes. Expression ratios for arf-1.1 and gst-22 genes were obtained by RT-qPCR. All experiments were repeated at least three times (biological replicates) and were internally controlled (technical replicate). Expression changes were obtained by calculating the relative expression levels using the 2−ΔΔCT method. See Materials and Methods for details. (DOCX) [file pone.0052493.s011.docx]

|  | CNCM I-3690/CNCM I-4317 | | CNCM I-3690/OP50 | |
| --- | --- | --- | --- | --- |
|  | Microarray | RT-qPCR | Microarray | RT-qPCR |
| arf-1.1 | 3,6 | 1,4 | 2,9 | 5,9 |
| gst-22 | 3,4 | 1,3 | 1,7 | 2,3 |

|  | p Paired t test |
| --- | --- |
| CNCM I-3690 vs CNCM I-4317 | 0,0067 |
| CNCM I-3690 vs OP50 | 0,0001 |

|  | p Paired t test |
| --- | --- |
| CNCM I-3690 vs CNCM I-4317 | 0,0021 |
| CNCM I-3690 vs OP50 | 0,0001 |
